# On the Scientific Investigation of Sanitary Questions

**Published:** 1855

**Authors:** C. Girdlestone


### ON THE SCIENTIFIC INVESTIGATION OF SANITARY QUESTIONS.

By the Rev. C. GIRDLESTONE, M.A.

I HAIL, with no common satisfaction, the commencement of the QUARTERLY JOURNAL OF PUBLIC HEALTH, under the auspices announced in the prospectus of the journal; since I feel certain that the great cause of sanitary reform will never rest on a secure foundation, without a thorough scientific investigation of certain elementary problems, on which practical men are at present more or less in the dark. Having taken part in the sanitary movement, almost from its commencement, and being more than ever convinced, the more I reflect on it, that it is pregnant with the most important advantages to the human race, in every point of view,—social, moral, and religious,—I may perhaps be allowed to suggest some of the difficulties that beset it, when reduced to practice, and which are now awaiting for a solution, which only science is competent to give.

It is certain, that practically the atmosphere is the chief vehicle of those noxious influences, whencesoever arising, which it is the business of the sanitary reformer to obviate or remove. Dampness and filthiness, putrescent effluvia, the exhalations of disease, and the refuse matters which the lungs and the skin of the most healthy are continually throwing off,—all these reach the human system for harm, by being wafted to and fro in the air. Ventilation, therefore, is the great object to be achieved. But how to ventilate? This, in most cases, is far from being an easy matter. How to secure for the lungs air ever fresh, and pure, and cool, without injury to the system through the skin, which is almost as abhorrent of a chilling draught as the lungs are of an adulterated atmosphere. This is one phase of the difficulty. But even if there were no risk of injury from cold to the skin, another difficult question would arise, viz., how to secure, within four walls, a ceiling, and a floor, as much pure air as the lungs of the inmates may require. And why is it that this cannot, as things now stand, be accomplished with any degree of certainty? Why do sanitary doctors differ so widely in their prescriptions? Why has such scandal to the cause of ventilation arisen from the great experiment pursued, with so little limit to expense, in the new Palace at Westminster? Because science has not yet determined, with sufficient clearness for our purpose, the law of diffusion of gases, nor drawn, with sufficient accuracy, the line of demarcation between their mechanical admixture, and their chemical combination. The air which we respire, when deprived of its oxygen, and laden with carbon, and at the same time altered in temperature, is it heavier than before, or lighter? Does it go upwards at once, or downwards? How does it intermingle itself amongst the air it comes in contact with in either case? How far are these processes affected by the temperature of the apartment, and by the nature of the various gases with which the pure air is already diluted? Which of these tend to neutralise, and which to aggravate, the noxious influences of the other; and under what circumstances are they either separable, or apt to combine? These and many like questions need to be determined far more accurately than hitherto they have been, with a special view to the exigencies of sanitary architecture. They need to be reduced to simple practical rules, for the use of those who know nothing of physical science; so as to shew plainly to the architect and the builder where and how to admit fresh air, and what provision to make for getting rid of that which is foul, much in the same way that any ordinary ship's mate, who may be altogether ignorant of astronomy, is enabled to profit by the scientific labours of astronomers, in his business of navigating the seas.

I will name another point wherein science might at once secure help in its own researches, and render the utmost service to those who are engaged in the details of sanitary reform. We want an instrument adapted to indicate the purity of the atmosphere in any definite place, or part of a room, and the degrees of its deterioration from time to time;—just as a thermometer shews its warmth, or a barometer its weight. They who first devised these notable instruments little thought of the extent to which they would be applied, in facilitating the progress of science and of the arts. Let Chemistry straightway furnish Sanitation with an instrument as serviceable in its way, wherewith to gauge the depths of atmospheric impurities, and to register them at any given time, if required; one that may become a household instrument in general use, wherewith to prove, beyond all dispute, how abominably our due allowance of vital air, as oxygen was once emphatically called, is often curtailed, if not absolutely poisoned. And, incidentally, one great benefit would be sure to result from the introduction of any such an instrument;—it would diffuse far and wide an interest in these questions, and an acquaintance with sanitary principles; just as the variations in the weight and temperature of air become matter of familiar observation, wherever there are a barometer and a thermometer.

The degree to which our higher classes are as yet ignorant of physical science, in its application to health, has been painfully made manifest on a late occasion. One official personage was asked whether it was true that a hospital for troops, with 3000 beds, was about to be established at Smyrna, and whether Government were aware that Smyrna is reputed the most unhealthy place in the Levant? The reply, according to the newspaper reporters of debates, a reply, by the way, which no one gainsaid, was to this effect:—"That the arrangements inside the hospital would be such as to render its healthiness independent of the atmosphere without!"

It may seem derogatory to the dignity of a scientific journal, for me to express a hope, that it may tend to apprise men in office of the fact, that the air outside a building has a great deal to do with that which is within, and to convince them that the convalescence of the wounded and the sick depends quite as much on a favourable locality as on doctors and nurses, instruments and drugs. And yet I hope I may be allowed to state my satisfaction in witnessing the commencement of a Health Journal at a period when the nation, smarting under its recent painful losses, may be better disposed than heretofore to believe, as it has been told on authority little short of demonstration for many years past, not only that preventible disease slays its many thousands at home in peace, whilst war slays only hundreds in proportion, but also, that in warfare health is strength, that we need to guard against disease no less diligently than against the enemy, and that, without due provision for the maintenance of the health of our armies, we may even while winning battles lose credit and character, and put in jeopardy the attainment of an honourable peace.

KINGSWINFORD RECTORY, DUDLEY,
*March* 14*th*, 1855.

